# Comparative effectiveness of abatacept versus TNF inhibitors in rheumatoid arthritis patients who are ACPA and shared epitope positive

**DOI:** 10.1186/s42358-024-00352-4

**Published:** 2024-01-19

**Authors:** Leslie R. Harrold, Keith Wittstock, Sheila Kelly, Xue Han, Joe Zhuo, Amy Schrader, Nicole Middaugh, Page C. Moore, Vadim Khaychuk

**Affiliations:** 1 CorEvitas, LLC, 350 5th Avenue, Waltham, MA 02451 USA; 2 University of Massachusetts Medical School, Worcester, MA USA; 3 Bristol Myers Squibb, Princeton, NJ USA

**Keywords:** Rheumatoid arthritis, TNF inhibitors, Epitopes, Registries, Abatacept

## Abstract

**Background:**

The *HLA-DRB1* shared epitope (SE) is a risk factor for the development of rheumatoid arthritis (RA) and the production of anti-citrullinated protein antibodies (ACPAs) in RA patients. Our objective was to examine the real-world effectiveness of abatacept versus tumor necrosis factor inhibitors (TNFi) in patients with RA who were SE and anti-cyclic citrullinated peptide antibody (anti-CCP3) positive.

**Methods:**

Abatacept or TNFi initiators who were SE + and anti-CCP3+ (> 20 U/mL) at or prior to treatment and had moderate or high CDAI score (> 10) at initiation were identified. The primary outcome was mean change in CDAI score over six months. Analyses were conducted in propensity score (PS)-trimmed and -matched populations overall and a biologic-experienced subgroup. Mixed-effects models were used.

**Results:**

In the overall PS-trimmed (abatacept, *n* = 170; TNFi, *n* = 157) and PS-matched cohorts (abatacept, *n* = 111; TNFi, *n* = 111), there were numerically greater improvements in mean change in CDAI between abatacept and TNFi but were not statistically significant. Similar trends were seen for biologic-experienced patients, except that statistical significance was reached for mean change in CDAI in the PS-trimmed cohort (abatacept, 12.22 [95% confidence interval (95%CI) 10.13 to 14.31]; TNFi, 9.28 [95%CI 7.08 to 11.48]; *p* = 0.045).

**Conclusion:**

In this real world cohort, there were numerical improvements in efficacy outcomes with abatacept over TNFi in patients with RA who were SE + and ACPA+, similar to results from a clinical trial population The only statistically significant finding after adjusting for covariates was greater improvement in CDAI with abatacept versus TNFi in the bio-experienced PS-trimmed cohort..

**Supplementary Information:**

The online version contains supplementary material available at 10.1186/s42358-024-00352-4.

## Introduction

Rheumatoid arthritis (RA) is a systemic autoimmune disease associated with a substantial impact on quality of life, daily functioning, morbidity, and mortality. The etiology of RA development has not been fully elucidated, however, studies in recent years suggest that RA arises due to a combination of genetic, epigenetic, and environmental components, including cigarette smoke [1, 2] and occupational dust exposure (e.g., silica) among others [3, 4]. Rheumatoid arthritis can be subclassified into seropositive and seronegative RA, defined by the presence or absence of anti-citrullinated protein antibodies (ACPA), respectively. Often years before clinical symptoms present, the presence of ACPA, rheumatoid factor (RF), and rising levels of C-reactive protein (CRP) in patients indicates that the relevant immune responses for RA development are initiated [1, 2]. 

Data from clinical trials [5–8] and real-world data [9–13] have consistently shown an association between abatacept treatment response and antibody seropositivity status. Recent studies have shown ACPA seropositivity is associated with differential response (DR) to biologic agents, with seropositive abatacept patients exhibiting a greater response than seronegative patients [14, 15]. However, a greater response was not seen among tumor necrosis factor inhibitors (TNFi) patients who were seropositive compared to seronegative [10, 12]. 

The most important genetic risk factor for RA, shared epitope (SE) alleles, reside in the major histocompatibility complex (MHC) Class II region and express human leukocyte antigen class II molecules encoded by DR (HLA-DR) [1, 2]. Previous research indicates many patients with RA who are seropositive for anti-citrullinated protein antibodies (ACPAs) have the presence of a shared epitope (SE) on the HLA-DRB1 allele [1, 16, 17]. A deeper understanding of the contribution of HLA-DRB1 to RA pathogenesis is essential in exploring the multiple interconnected contributing factors such as genetic, epigenetic, and environmental exposures that lead to RA morbidity [1]. 

Current treatment recommendation for RA focus on the treat to target approach, specifically escalating care based on disease activity. The most recent 2021 RA recommendations mention the use of biologic disease-modifying antirheumatic drugs (bDMARDs) and targeted synthetic (tsDMARDs) when patients are not at target disease activity despite an adequate trial of methotrexate [18]. However, choice of individual b/tsDMARDs is a trial and error approach. In an ideal world, clinicians would tailor therapy to the specific characteristics of patients (i.e., precision medicine) to achieve disease remission as soon as possible, considering that delays in achieving disease control are associated with worse outcomes [15]. 

While we have previously compared the effectiveness of abatacept versus TNFi in anti-cyclic citrullinated peptide antibody positive (anti-CCP+) RA patients, [12] to our knowledge, this is the first study to compare treatment responses within a subset of real-world patients with a genetic marker of the disease. Therefore, the aim of this study focused on understanding whether the presence of a SE on the HLA-DRB1 allele is related to a differential response to abatacept or TNFi medications; specifically, to compare outcomes in anti-CPP + RA patients treated with abatacept versus TNFi in those with the SE.

## Methods

### Study sample

#### Registry overview

The CorEvitas RA Registry is an ongoing longitudinal observational clinical registry that was established in 2001. As of March 31, 2022, the Registry included 217 private and academic active clinical sites with 931 participating rheumatologists throughout 42 states in the U.S. The mean duration of patient follow-up is 4.7 years (median 3.4 years). Data are collected using CorEvitas questionnaires from patients and providers during routine office visits.

#### Overall CERTAIN population

For this study, we used patients enrolled in CorEvitas’ Comparative Effectiveness Registry to Study Therapies for Arthritis and Inflammatory Conditions (CERTAIN; ClinicalTrials.gov identifier: NCT01625650) study, which was a substudy of the CorEvitas RA Registry that mandated regular data collection at 3-month intervals, with uniform collection of blood samples for all patients initiating biologic treatment. Patients’ baseline characteristics and blood samples were collected within 72 h of the drug initiation. CorEvitas’ CERTAIN substudy included patients with RA who were ≥ 18 years of age, had moderate or high disease activity (e.g., Clinical Disease Activity Index [CDAI] score > 10) at enrollment and either started bDMARD therapy or switched from a prior bDMARD TNFi (e.g., adalimumab, etanercept, certolizumab pegol, golimumab, and infliximab) or non-TNFi bDMARD (e.g., abatacept, rituximab, and tocilizumab). Patients were followed for 12 months or until they switched/discontinued bDMARD therapy. CERTAIN patients were recruited from 43 private practices and academic sites with 117 participating rheumatologists. Patient enrollment in the CERTAIN substudy began in November of 2010, recruitment was completed in April 2014, and patient follow-up was completed in May 2015. CERTAIN was comprised of 2,795 initiations by the 2,350 patients who completed enrollment [19]. 

#### Analysis population

Abatacept and TNFi initiators 18 years of age or older from CERTAIN were required to have the presence of a SE on the HLA-DRB1 allele (SE+). In addition, patients had to have a moderate to high CDAI (CDAI > 10) and be anti-CCP3+ (CCP3 > 20 U/mL) at initiation, with initiation defined as first-time use of the specific drug; initiations may have been as monotherapy or in combination with conventional synthetic (cs) DMARDs. For TNFi initiators, there could be no prior exposure to abatacept in an attempt to identify a cohort of patients who would be just as likely to initiate a TNFi as compared to Abatacept. All patients had a 6-month follow-up visit after initiation (+/- 3 months) or stopped the drug prior to three months.

#### Propensity score trimming and matching

A propensity score (PS) model was used to identify patients with similar disease activity and similar number of prior biologics at baseline to determine which DNA samples to send for genotyping. Using a logistic regression model with abatacept initiation as the outcome, a PS was estimated using patient baseline characteristics with a standardized difference (sDiff) > 0.1 stratified by prior number of biologics: no prior biologics, 1 prior biologic, and > 1 prior biologic. Using the PS, abatacept and TNFi initiations with overlapping PS within each stratum were selected for further study. DNA samples for 228 abatacept and 240 TNFi initiations were sent for genotyping.

Among patients identified with the presence of the SE, trimming based on comparing the distribution of propensity scores between abatacept and TNFi SE initiators within line of therapy was performed, and the observations that did not fall within the overlapping range were excluded from the study cohort. Baseline characteristics included in the PS model included age, work status, smoking status, CDAI, current MTX use and modified Charlson comorbidity index; for no and 1 prior biologic only– education (college or above), BMI, history of hypertension; for no prior biologic only– duration of RA, DAS28-CRP and current Prednisone use; for 1 and > 1 prior biologic only– RF titer and patient reported pain; for 1 prior biologic only– gender; for no and > 1 prior biologic only– number of prior csDMARDs; for > 1 prior biologic only– mHAQ and CRP.

Using the PS developed for the trimmed cohort, TNFi initiators were matched to abatacept initiators within each line of therapy using 1:1 PS matching.

### Outcome measures

Data were collected during the study period from physician and patient questionnaires completed during clinical encounters associated with the CERTAIN trial. These forms were used to gather information on disease severity and activity; comorbidities; use of medications including steroids, csDMARDs, tsDMARDs and bDMARDs; and adverse events. Data elements collected included swollen joint count in 28 joints, tender joint count in 28 joints, and the Physician Global Assessment (PGA) as measured by a visual analog scale (VAS) scores 0-100 [20]. Patient-reported outcomes included Patient Global Assessment (PtGA) (VAS 0-100), patient-reported pain (VAS 0-100), patient-reported fatigue (VAS 0-100), and the modified Health Assessment Questionnaire (mHAQ) assessing physical function [21]. Data on demographics, RA disease characteristics, and disease activity were available for > 98% of patients.

#### Primary and secondary outcomes

The primary outcome was mean change in CDAI score over six months following initiation. Secondary outcomes at six months included achievement of remission (CDAI ≤ 2.8), achievement of low disease activity (LDA) (CDAI score ≤ 10); mean change from baseline in patient-reported pain (VAS 0-100), fatigue (VAS 0-100), and Patient Global Assessment (PtGA; VAS 0-100); and mHAQ. For patients with a 6-month follow-up visit after initiation (+/-3 months), response was indicated by disease activity reported at that visit. As sensitivity analyses, the outcomes were re-evaluated in both PS-trimmed and -matched cohorts among only those who were biologic-experienced.

#### Switching Status

Switching outcomes of interest included percent of patients who remained on drug at the 6-month visit; percent who discontinued initiated drug and did not start another biologic at/before the 6-month visit; and the percent who switched initiated drug to another biologic at/before the 6-month visit. Switching status was compared between the treatment cohorts. For patients stopping or switching treatment prior to six months, 6-month responses for continuous outcomes were reported using the response reported at their last CERTAIN visit prior to stopping/switching therapy, and dichotomous outcomes were imputed as non-response.

### Statistical analyses

Baseline characteristic comparisons were made between treatment groups within line of therapy in which the first line includes biologic-naïve patients starting their first biologic, the second line includes patients with one prior biologic, and the third + line includes patients with two or more prior biologics.

Standardized differences were considered after comparing the study population to evaluate the balance between abatacept and TNFi patient who were SE+. A sDiff that is less than 0.1 indicates a negligible difference between treatment groups; therefore, variables with sDiff greater than 0.1 between the groups were considered to have remained potentially unbalanced [22]. 

Analyses were performed using both propensity score (PS) trimmed and PS matched approaches in the overall population with sensitivity analyses among biologic-experienced patients (overall trimmed, overall matched, biologic-experienced trimmed, biologic-experienced matched). The PS trimmed cohort maximizes the sample size while excluding patients who are not comparable to the other treatment group. The PS matched analyses maximize the comparability of the two treatment groups but sacrifices sample size and thus reducing the statistical power. Mixed-effects models (with site ID and patient ID as the random factors with patient nested within site) were used. The primary fixed effect of interest was treatment group (using abatacept initiators as the treatment group). The unit of analysis was an initiation of a biologic agent. Potential adjustments were investigated and incorporated into the model including *a priori factors* (e.g., baseline CDAI score, number of prior biologics used, modified Charlson comorbidity index, and current methotrexate use) thought to influence medication response and factors where there was a residual imbalance between the treatment groups. After PS- trimming and -matching, the remaining imbalanced variables were included as covariates in the multivariable models. A *p*-value < 0.05 was considered statistically significant. All statistical analyses were performed using (Stata Version 15.0, College Station, TX).

### Ethics

The study was performed following the Declaration of Helsinki and the Guidelines for Good Pharmacoepidemiology Practice (GPP). All participating investigators were required to obtain full board approval for conducting noninterventional research involving human subjects with a limited dataset. Sponsor approval and continuing review was obtained through a central Institutional Review Board (IRB), the New England Independent Review Board (NEIRB; no. 120160610). For academic investigative sites that did not receive authorization to use the central IRB, full board approval was obtained from their respective governing IRBs, and documentation of approval was submitted to CorEvitas, LLC before the site’s participation and the initiation of any study procedures. All patients in the registry were required to provide written informed consent and authorization before participating.

## Results

Among the 2,795 biologic initiations in the CERTAIN registry, there were 531 abatacept and 1,656 TNFi initiations, of which there were 266 abatacept and 895 TNFi initiations who were anti-CCP3+ (CCP3 > 20 U/ml) at time of initiation. Among those, there were 239 abatacept and 709 TNFi initiations with at least a moderate CDAI (CDAI > 10), available DNA sample, and no prior history of abatacept. A propensity score model was used to identify initiations similar at baseline to select which DNA samples to send for shared epitope testing; 228 abatacept and 240 TNFi initiations were selected. After the DNA samples were genotyped, 178 abatacept and 195 TNFi initiations were identified as having the SE and used in this study (Supplemental Fig. 1).

### Outcomes in the overall PS-trimmed and -matched cohorts

The baseline characteristics prior to propensity score trimming (Supplemental Table S1) showed that the two treatment cohorts differed (absolute value of the sDiff > 0.10) in terms of gender, race, insurance status, working status, comorbidities, duration of RA, disease activity, and prior treatment of RA. After PS-trimming, the overall population included 170 abatacept initiators and 157 TNFi initiators. Among the overall PS-trimmed population of 170 abatacept and 157 TNFi initiators, baseline characteristics that remained imbalanced (sDiff > 0.10) between biologic treatments included sex, RF+, physician and patient global assessments, pain, fatigue, and prior use of treatments for RA. Abatacept patients were more often female, RF + with longer disease duration, worse baseline patient-reported measures, and received their medication later in the line of therapy. Baseline characteristics in the overall PS-matched cohort, which included 111 pairs of abatacept and TNFi initiators, were generally well balanced. Of these pairs, 19 were biologic-naïve, 49 had used one prior biologic, and 43 had used ≥ 2 prior biologics (Table 1). Similar trends for imbalances in baseline characteristics were seen in the biologic-experienced cohort (PS-trimmed; abatacept, *n* = 145, TNFi, *n* = 129; PS-matched; abatacept, *n* = 92, TNFi, *n* = 92) (Table 2).

**Table 1:** Baseline characteristics in the overall population

	Overall Population					
	PS-Matched			PS-Trimmed		
At time of initiation	Abatacept *N* = 111	TNF *N* = 111	Standardized Difference	Abatacept *N* = 170	TNF *N* = 157	Standardized Difference
Age (years), Mean (SD)	56.2 (12.5)	57.6 (12.3)	0.11	56.6 (12.9)	56.9 (12.4)	0.03
Female, *n* (%)	82 (73.9)	81 (73.0)	0.02	126 (74.1)	108 (68.8)	0.12
White, *n* (%)	107 (98.2)	99 (90.0)	0.35	166 (98.8)	143 (92.9)	0.30
Medicaid, *n* (%)	9 (8.1)	7 (6.3)	0.07	15 (8.8)	10 (6.4)	0.09
Full/Part time work, *n* (%)	54 (48.7)	55 (49.6)	0.02	85 (50.0)	75 (47.8)	0.04
Retired, *n* (%)	23 (20.7)	27 (24.3)	0.09	32 (18.8)	41 (26.1)	0.17
Disabled, *n* (%)	18 (16.2)	21 (18.9)	0.07	34 (20.0)	27 (17.2)	0.07
Never smoker, *n* (%)	42 (37.8)	43 (38.7)	0.02	69 (40.6)	63 (40.1)	0.01
Former smoker, *n* (%)	41 (36.9)	38 (34.2)	0.06	58 (34.1)	58 (36.9)	0.06
Current smoker, *n* (%)	28 (25.2)	30 (27.0)	0.04	43 (25.3)	36 (22.9)	0.06
BMI, Obese (≥ 30), *n* (%)	42 (37.8)	34 (30.6)	0.15	67 (39.4)	55 (35.0)	0.09
Hypertension, *n* (%)	37 (33.3)	38 (34.2)	0.02	60 (35.3)	50 (31.9)	0.07
Prior serious Infections, *n* (%)	6 (5.4)	6 (5.4)	0.00	11 (6.5)	8 (5.1)	0.06
Cardiovascular, *n* (%)	18 (16.2)	18 (16.2)	0.00	26 (15.3)	24 (15.3)	0.00
Duration of RA year, Mean (SD)	10.5 (10.6)	11.4 (10.8)	0.09	11.4 (10.2)	10.4 (10.8)	0.10
CDAI, Mean (SD)	27.5 (12.4)	27.2 (11.6)	0.03	27.8 (11.8)	27.5 (11.9)	0.02
TJC, Mean (SD)	9.6 (6.4)	9.4 (6.9)	0.03	9.9 (6.3)	9.8 (7.0)	0.01
SJC, Mean (SD)	7.6 (5.4)	7.4 (4.5)	0.03	7.7 (5.4)	7.7 (4.9)	0.00
CRP, Mean (SD)	10.5 (15.2)	10.4 (14.6)	0.01	10.0 (14.6)	10.7 (15.1)	0.05
DAS28-CRP, Mean (SD)	4.7 (1.1)	4.7 (1.0)	0.03	4.7 (1.1)	4.7 (1.0)	0.05
RF, Mean (SD)	203.7 (283.0)	177.4 (260.8)	0.10	192.3 (262.9)	180.5 (247.5)	0.05
RF+, *n* (%)	99 (89.2)	99 (89.2)	0.00	155 (91.2)	138 (87.9)	0.11
mHAQ, Mean (SD)	0.5 (0.5)	0.5 (0.5)	0.01	0.5 (0.5)	0.5 (0.5)	0.05
PGA (VAS 0-100), Mean (SD)	49.8 (21.0)	51.3 (17.4)	0.08	48.3 (19.5)	50.8 (18.9)	0.13
PtGA (VAS 0-100), Mean (SD)	53.0 (23.4)	52.0 (23.8)	0.05	54.0 (23.5)	49.9 (24.8)	0.17
Pain (VAS 0-100), Mean (SD)	53.1 (23.9)	53.0 (27.2)	0.00	54.8 (24.4)	51.2 (28.3)	0.14
Fatigue (VAS 0-100), Mean (SD)	55.5 (28.3)	54.1 (29.5)	0.05	56.1 (27.7)	51.9 (29.4)	0.15
Biologic Naïve, *n* Yes, *n* (%)	111 19 (17.1)	111 19 (17.1)	0.00	170 25 (14.7)	157 28 (17.8)	0.08
Prior csDMARD usage count, Mean (SD)	1.1 (1.1)	1.2 (1.2)	0.09	1.3 (1.3)	1.1 (1.2)	0.17
Prior TNFi usage count, *n*	111	111		170	157	
0, *n* (%)	20 (18.0)	21 (18.9)	0.02	26 (15.3)	31 (19.8)	0.12
1, *n* (%)	53 (47.8)	50 (45.1)	0.05	60 (35.3)	84 (53.5)	0.37
2+, *n* (%)	38 (34.2)	40 (36.0)	0.04	84 (49.4)	42 (26.8)	0.48
Prior non-TNFi usage count, *n*	111	111		170	157	
0, *n* (%)	99 (89.2)	105 (94.6)	0.20	148 (87.1)	148 (94.3)	0.25
1, *n* (%)	10 (9.0)	6 (5.4)	0.14	18 (10.6)	9 (5.7)	0.18
2+, *n* (%)	2 (1.8)	0 (0.0)	0.19	4 (2.4)	0 (0.0)	0.22
Prior biologic usage count, *n*	111	111		170	157	
0, *n* (%)	19 (17.1)	19 (17.1)	0.00	25 (14.7)	28 (17.8)	0.08
1, *n* (%)	49 (44.1)	49 (44.1)	0.00	54 (31.8)	83 (52.9)	0.44
2, *n* (%)	28 (25.2)	37 (33.3)	0.18	57 (33.5)	38 (24.2)	0.21
3+, *n* (%)	15 (13.5)	6 (5.4)	0.28	34 (20.0)	8 (5.1)	0.46
Current Prednisone use, *n*	111	111		170	157	
Yes, *n* (%)	52 (46.9)	44 (39.6)	0.15	74 (43.5)	67 (42.7)	0.02
Prednisone dose, *n*	52	43		73	66	
Mean (SD)	7.6 (4.6)	7.3 (4.2)	0.07	7.4 (5.0)	7.4 (3.9)	0.01
MTX dose, *n*	54	56		87	83	
Mean (SD)	17.4 (5.3)	17.4 (4.7)	0.00	17.8 (5.2)	17.7 (4.9)	0.02
Mono and Combo Therapy						
Monotherapy	39 (35.1)	38 (34.2)	0.06	56 (32.9)	51 (32.5)	0.07
Combo with MTX	44 (39.6)	47 (42.3)		73 (42.9)	72 (45.9)	
Combo with non-MTX csDMARD	17 (15.3)	16 (14.4)		26 (15.3)	22 (14.0)	
Combo with MTX & non-MTX csDMARD	11 (9.9)	10 (9.0)		15 (8.8)	12 (7.6)	

A standardized difference that is less than 0.10 indicates a negligible difference between treatment groups; therefore, variables with standardized differences greater than 0.10 between the groups were considered to have remained potentially unbalanced; BMI, body mass index; CDAI, Clinical Disease Activity Index; SD, standard deviation; TJC(28), Tender 28-Joint Count; SJC(28), Swollen 28-Joint Count; CRP, C-reactive protein; DAS28-CRP, Disease Activity Score-28 using CRP; RF, rheumatoid factor; mHAQ, modified Health Assessment Questionnaire; PGA, physician global assessment; PtGA, patient global assessment; VAS, visual analogue scale; csDMARD, conventional synthetic disease modifying anti-rheumatic drug; TNFi, tumor necrosis factor inhibitor; MTX, methotrexate

**Table 2:** Baseline characteristics of the biologic-experienced population

	Biologic -Experienced					
	PS-Matched			PS-Trimmed		
At time of initiation	Abatacept *N* = 92	TNF *N* = 92	Standardized Difference	Abatacept *N* = 145	TNF *N* = 129	Standardized Difference
Age (years), Mean (SD)	56.2 (12.5)	56.8 (12.2)	0.05	56.0 (12.6)	56.5 (12.4)	0.04
Female, *n* (%)	65 (70.7)	67 (72.8)	0.05	107 (73.8)	87 (67.4)	0.14
White, *n* (%)	89 (98.9)	82 (90.1)	0.39	142 (99.3)	117 (92.9)	0.34
Medicaid, *n* (%)	8 (8.70)	7 (7.6)	0.04	14 (9.7)	9 (7.0)	0.10
Full/Part time work, *n* (%)	47 (51.1)	49 (53.3)	0.04	78 (53.8)	65 (50.4)	0.07
Retired, *n* (%)	19 (20.7)	20 (21.7)	0.03	24 (16.6)	32 (24.8)	0.20
Disabled, *n* (%)	15 (16.3)	18 (19.6)	0.08	31 (21.4)	23 (17.8)	0.09
Never smoker, *n* (%)	34 (37.0)	34 (37.0)	0.00	59 (40.7)	50 (38.8)	0.04
Former smoker, *n* (%)	33 (35.9)	30 (32.6)	0.07	46 (31.7)	46 (35.7)	0.08
Current smoker, *n* (%)	25 (27.2)	28 (30.4)	0.07	40 (27.6)	33 (25.6)	0.05
BMI, Obese (≥ 30), *n* (%)	35 (38.0)	31 (33.7)	0.09	57 (39.3)	50 (38.8)	0.01
Hypertension, *n* (%)	30 (32.6)	32 (34.8)	0.05	50 (34.5)	41 (31.8)	0.06
Serious Infections, *n* (%)	6 (6.5)	6 (6.5)	0.00	11 (7.6)	7 (5.4)	0.09
Cardiovascular, *n* (%)	15 (16.3)	13 (14.1)	0.06	22 (15.2)	17 (13.2)	0.06
Duration of RA years, Mean (SD)	11.1 (10.3)	11.5 (10.4)	0.03	11.9 (9.9)	10.9 (10.6)	0.10
CDAI, Mean (SD)	27.6 (13.0)	27.4 (12.1)	0.02	28.2 (12.3)	27.7 (12.4)	0.04
TJC(28), Mean (SD)	9.8 (6.6)	9.4 (7.1)	0.05	10.2 (6.5)	9.7 (7.2)	0.07
SJC(28), Mean (SD)	7.7 (5.6)	7.7 (4.6)	0.00	7.8 (5.6)	8.0 (5.2)	0.04
CRP, Mean (SD)	10.3 (14.8)	9.2 (14.1)	0.07	9.7 (14.4)	10.2 (15.3)	0.04
DAS28-CRP, Mean (SD)	4.7 (1.2)	4.6 (1.1)	0.06	4.7 (1.1)	4.6 (1.0)	0.09
RF, Mean (SD)	210.7 (258.0)	163.0 (244.4)	0.19	195.9 (245.4)	175.5 (236.7)	0.08
RF+, *n* (%)	85 (92.4)	81 (88.0)	0.15	135 (93.1)	113 (87.6)	0.19
mHAQ, Mean (SD)	0.5 (0.5)	0.5 (0.5)	0.00	0.6 (0.5)	0.5 (0.5)	0.10
PGA (VAS 0-100),						
Mean (SD)	49.9 (21.7)	51.2 (17.5)	0.07	48.7 (19.8)	50.5 (19.2)	0.10
PtGA (VAS 0-100),						
PGA, Mean (SD)	51.4 (24.1)	50.8 (23.6)	0.03	53.4 (24.4)	49.4 (24.5)	0.16
Pain (VAS 0-100),						
Mean (SD)	53.2 (25.0)	52.5 (27.1)	0.03	56.0 (25.1)	51.2 (28.3)	0.18
Fatigue (VAS 0-100),						
Mean (SD)	56.0 (28.7)	53.5 (29.0)	0.09	57.6 (27.6)	52.2 (28.9)	0.19
Prior csDMARD usage count,						
Mean (SD)	1.2 (1.2)	1.3 (1.3)	0.09	1.4 (1.3)	1.2 (1.2)	0.18
Prior TNFi usage count, *n*	92	92		145	129	
0, *n* (%)	1 (1.1)	2 (2.2)	0.09	1 (0.7)	3 (2.3)	0.13
1, *n* (%)	53 (57.6)	50 (54.4)	0.07	60 (41.4)	84 (65.1)	0.49
2+, *n* (%)	38 (41.3)	40 (43.5)	0.04	84 (57.9)	42 (32.6)	0.53
Prior non-TNFi usage count, *n*	92	92		145	129	
0, *n* (%)	80 (87.0)	86 (93.5)	0.22	123 (84.8)	120 (93.0)	0.26
1, *n* (%)	10 (10.9)	6 (6.5)	0.15	18 (12.4)	9 (7.0)	0.18
2+, *n* (%)	2 (2.2)	0 (0.0)	0.21	4 (2.8)	0 (0.0)	0.24
Prior biologic usage count, *n*	92	92		145	129	
0, *n* (%)	0 (0.0)	0 (0.0)	N/A	0 (0.0)	0 (0.0)	N/A
1, *n* (%)	49 (53.3)	49 (53.3)	0.00	54 (37.2)	83 (64.3)	0.56
2, *n* (%)	28 (30.4)	37 (40.2)	0.20	57 (39.3)	38 (29.5)	0.21
3+, *n* (%)	15 (16.3)	6 (6.5)	0.31	34 (23.5)	8 (6.2)	0.50
Current MTX use, *n*	92	92		145	129	
Yes, *n* (%)	43 (46.7)	44 (47.8)	0.02	72 (49.7)	64 (49.6)	0.00
Current Prednisone use, *n*	92	92		145	129	
Yes, *n* (%)	43 (46.7)	37 (40.2)	0.13	64 (44.1)	55 (42.6)	0.03

^a^A standardized difference that is less than 0.10 indicates a negligible difference between treatment groups; therefore, variables with standardized differences greater than 0.10 between the groups were considered to have remained potentially unbalanced; BMI, body mass index; CDAI, Clinical Disease Activity Index; SD, standard deviation; TJC(28), Tender 28-Joint Count; SJC(28), Swollen 28-Joint Count; CRP, C-reactive protein; DAS28-CRP, Disease Activity Score-28 using CRP; RF, rheumatoid factor; mHAQ, modified Health Assessment Questionnaire; PGA, physician global assessment; PtGA, patient global assessment; VAS, visual analogue scale; csDMARD, conventional synthetic disease modifying anti-rheumatic drug; TNFi, tumor necrosis factor inhibitor; MTX, methotrexate

**Fig. 1:**
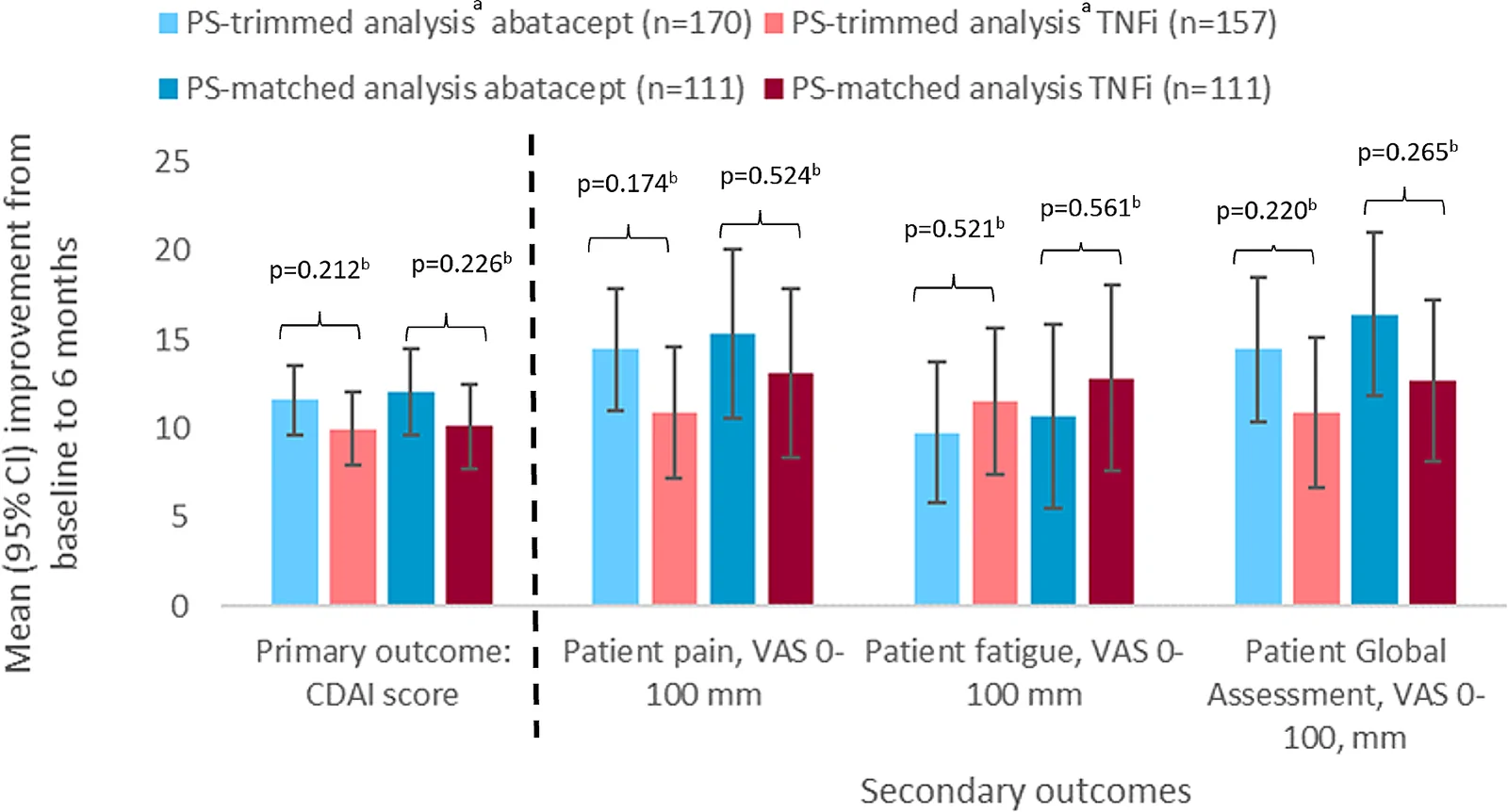
Primary and key secondary outcomes in the overall PS-trimmed and -matched populations

In the overall population (Fig. 1), for both the PS-trimmed and -matched cohorts, the mean change in CDAI from baseline to six months was greater for the abatacept group than the TNFi group, but it was not statistically significant in adjusted models (PS-trimmed: abatacept initiators, 11.61 [95% Confidence Interval (CI) 9.65 to 13.57]; TNFi initiators, 9.99 [95% CI 7.97 to 12.00]; *p* = 0.212; PS-matched: abatacept initiators, 12.10 [95% CI 9.67 to 14.53]; TNFi initiators, 10.12 [95% CI 7.74 to 12.51]; *p* = 0.226). Similarly, for the secondary outcomes of mean change in patient pain and PtGA, there were numerically greater improvements with abatacept than with TNFi, although not statistically significant. Conversely, mean change in patient fatigue was numerically greater with TNFi than abatacept. Compared with TNFi, the likelihood of achieving LDA or remission did not differ between patients treated with abatacept or TNFi, as the 95% CI crossed 1.0, indicating non-significance (PS-trimmed: abatacept initiators, LDA OR = 1.76, [95% CI 0.53 to 5.86] and remission OR = 1.40 [95% CI 0.56 to 3.53] and PS-matched: abatacept initiators, LDA OR = 1.55 [95%CI 0.36 to 6.64 and remission OR = 1.58 [95% CI 0.58, 4.34]). There was no statistically significant difference between treatments in the percentage of patients who remained on study medication at six months (Table 3).

**Table 3:** Additional secondary outcomes in the overall PS-trimmed and -matched populations

Outcome	PS-trimmed analysis^a^			PS-matched analysis		
	Abatacept (*n* = 170)	TNFi (*n* = 157)	*p* value^b^	Abatacept (*n* = 111)	TNFi (*n* = 111)	*p* value^b^
Response to therapy, Adjusted OR (95% CI)^c^						
Achievement of LDA	1.76 (0.53, 5.86)	Ref	0.36	1.55 (0.36, 6.64)	Ref	0.55
Achievement of remission	1.40 (0.56, 3.53)	Ref	0.47	1.58 (0.58, 4.34)	Ref	0.37
Drug retention						
Patients who remained on drug at 6-month visit, *n*(%)	117 (68.82)	101 (64.33)	0.39	79 (71.17)	73 (65.77)	0.39

^a^The PS-trimmed cohort included patients with scores overlapping both populations

^b^*p* values for secondary outcomes are from the adjusted mixed-effects models. Drug retention was assessed using the chi-square test

^c^OR (95% CI) for difference between treatments at 6-month visit was adjusted for covariates where standardized difference remained imbalanced

LDA = Low Disease Activity; OR = Odds Ratio; PS = Propensity Score; Ref = Reference; TNFi = TNF Inhibitor

Adjusted variables for overall PS-trimmed and PS-matched cohort included *a priori* covariates CDAI, number prior biologics used, modified Charlson comorbidity index and current MTX use, and covariates that remained imbalanced– work status; for PS-trimmed only– gender, body weight, age at RA onset, RF+, patient reported pain and fatigue, and number prior csDMARDs; for PS-matched only– age, BMI, and current Prednisone use

### Outcomes in the biologic-experienced PS-trimmed and -matched cohorts

**Fig. 2:**
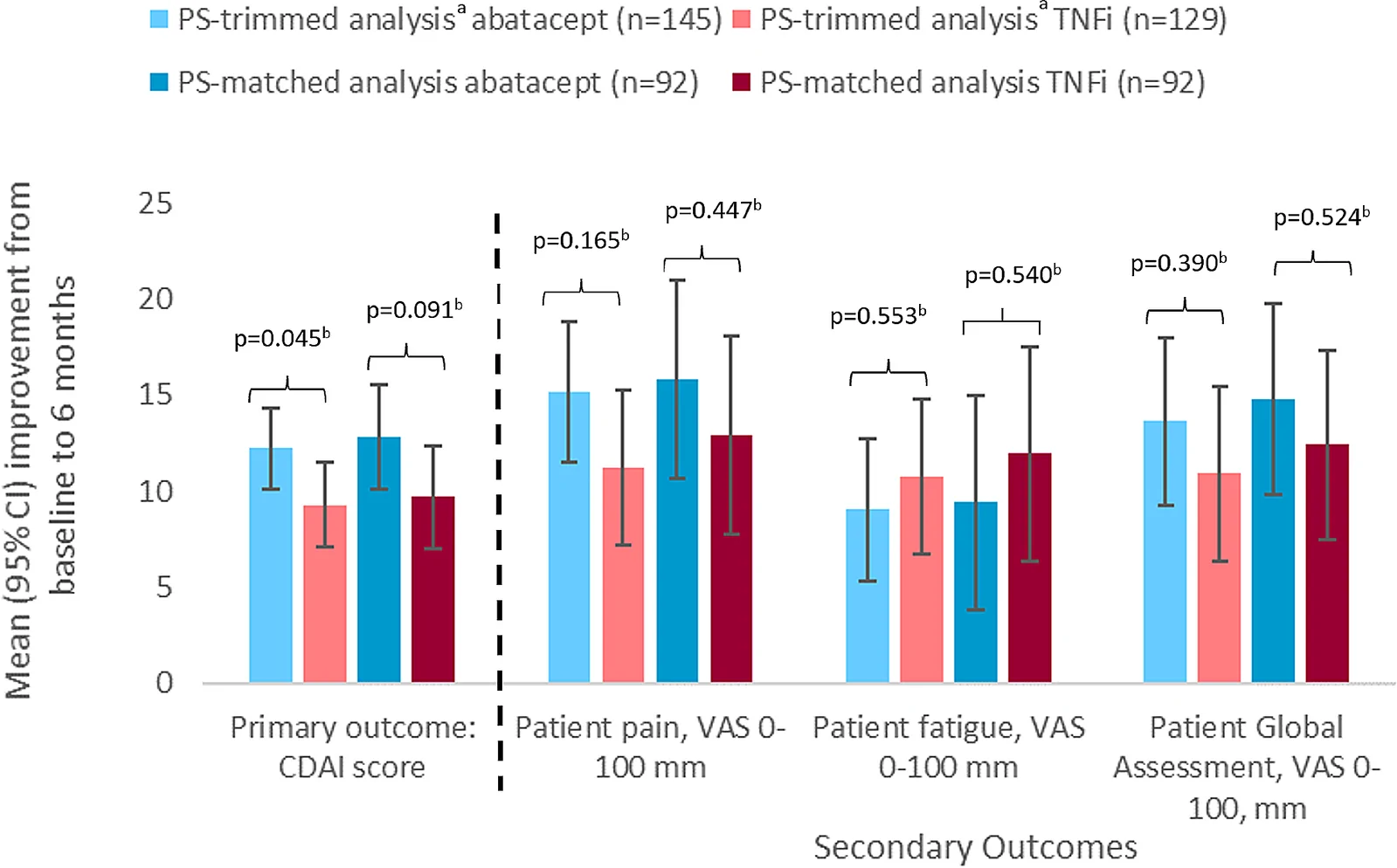
Primary and key secondary outcomes in the biologic-experienced PS-trimmed and -matched populations

In the biologic-experienced population, mean change in CDAI from baseline to six months was statistically significantly higher among patients receiving abatacept as compared to TNFi among the PS-trimmed cohort (145 abatacept and 129 TNFi) in adjusted models (abatacept initiators, 12.22 [95% CI 10.13 to 14.31]; TNFi initiators, 9.28 [95% CI 7.08 to 11.48]; *p* = 0.045). A similar trend was seen in the PS-matched cohort (92 abatacept and TNFi pairs), but it was not statistically significant (abatacept initiators, 12.84 [95% CI 10.12 to 15.56]; TNFi initiators, 9.69 [95% CI 7.03 to 12.35]; *p* = 0.091) (Fig. 2). Mean improvement in secondary outcomes of patient pain and PtGA showed greater numerical advantage to abatacept in the PS-trimmed and -matched cohorts and the likelihood of achieving LDA or remission did not differ between abatacept and TNFi treated patients. Trends for the secondary outcomes and switching status in biologic-experienced patients were similar to those seen for the overall cohort (Fig. 2; Table 4).

**Table 4:** Additional secondary outcomes in the biologic-experienced PS-trimmed and -matched populations

Outcome	PS-trimmed analysis^a^			PS-matched analysis		
	Abatacept (*n* = 145)	TNFi (*n* = 129)	*p* value^b^	Abatacept (*n* = 92)	TNFi (*n* = 92)	*p* value^b^
Response to therapy, Adjusted OR (95% CI)^c^						
Achievement of LDA	2.13 (0.51, 8.85)	Ref	0.30	1.87 (0.34, 10.31)	Ref	0.47
Achievement of remission	1.70 (0.52, 5.49)	Ref	0.38	2.08 (0.55, 7.89)	Ref	0.28
Drug retention						
Patients who remained on drug at 6-month visit, *n*(%)	99 (68.28)	82 (63.57)	0.41	65 (70.65)	60 (65.22)	0.43

^a^The PS-trimmed cohort included patients with scores overlapping both populations

^b^*p* values for secondary outcomes are from the adjusted mixed-effects models. Drug retention was assessed using the chi-square test

^c^OR (95% CI) for difference between treatments at 6-month visit was adjusted for covariates where standardized difference remained imbalanced

LDA = Low Disease Activity; OR = Odds Ratio; PS = Propensity Score; Ref = Reference; TNFi = TNF Inhibitor

Adjusted variables for biologic-experienced PS-trimmed and PS-matched cohort included *a priori* covariates CDAI, number prior biologics used, modified Charlson comorbidity index and current MTX use, and covariates that remained imbalanced– work status; for PS-trimmed only– gender, body weight, duration of RA, RF+, mHAQ, patient reported pain and fatigue, and number prior csDMARDs; for PS-matched only– BMI, RF titer and current Prednisone use

## Discussion

Understanding the contribution of HLA-DRB1 to RA pathogenesis and exploring the impact of SE on treatment outcomes may lead to new and advanced diagnostics and treatments for those who suffer from this incapacitating disease. This real-world analysis compared the clinical effectiveness of abatacept versus TNFi over six months in RA patients who were SE+. We used PS-trimming and -matching to reduce selection bias given differences in how these medications are utilized. While most comparisons were not statistically significant, we consistently found a greater numerical improvement in most efficacy outcomes with abatacept over TNFi in patients with long-standing RA who were SE + over six months in the overall population for both the PS-trimmed and -matched cohorts. Of note, these results are similar to a prior clinical trial [15]. A similar pattern was seen in the sensitivity analyses, which focused on a biologic-experienced cohort. However, there was a statistically significant advantage for use of abatacept over TNFi for mean improvement in CDAI in the biologic-experienced PS-trimmed cohort. Switching status was similar in both the PS-trimmed and -matched overall cohorts and those who were biologic-experienced. Given the lack of consistent statistically significant findings, further exploration is needed to confirm a potential relationship between SE + status and a differential response to medications.

While precision medicine has come to other medical conditions, there is an ongoing need in the management of RA to identify the right medications for the right patients at the right time. Of interest is whether the SE can be used to guide therapy. Given the known association between ACPAs and SE status, [1, 16, 17] it has been hypothesized the patients who are SE + will have an enhanced clinical response to treatment with a T-cell co-stimulatory modulator, such as abatacept, as abatacept blocks the interaction between CD80/CD86 on APCs and CD28 on T cells [15]. The Early AMPLE study (NCT02557100) found numerically higher efficacy responses among patients treated with abatacept versus adalimumab after 23 weeks of treatment. The response was more pronounced among SE + abatacept-treated patients although the differences were not statistically significant in this study with a limited sample size (*n* = 80) [15].

This study has many strengths. Of note, we were able to use the CERTAIN study, which included rich data on patient demographics, comorbidities, disease characteristics, disease activity, treatments, and biospecimens that allowed for genetic testing. In addition, our results were similar to what has been demonstrated by other authors using clinical trial populations. Finally, we used advanced epidemiological methods (e.g., PS-trimming and -matching) to reduce selection bias.

As with all studies, there are limitations as well. Comparing treatment selection in any real-world setting is not random as physicians are influenced by numerous factors when prescribing treatments, including individual patient profiles. However, to offset potential selection bias, we used both PS-trimming and -matching. There are advantages and disadvantages to using both the PS-trimmed and -matched cohorts. The trimmed cohort adjusts for patient differences and maximizes precision with a larger sample size and smaller standard errors; however, some residual confounding may exist. Further, the matched cohort loses power due to reduced sample size, but patients are well balanced with less bias. Due to the inclusion criteria of the study and the need for biospecimens for genetic testing, this did limit the number of patients that could be studied which may have impacted our ability to find differences. Further exploration in different cohorts, particularly in more diverse patient populations, should be pursued. In addition, we have the limited ability to explore the contribution of changing mechanism of action versus the direct effects of the respective drugs in an SE + population.

## Conclusions

Similar to what was found in a prior clinical trial population, there was a consistent numerical improvement (although most comparisons were not statistically significant) in most efficacy outcomes with abatacept over TNFi in patients with long-standing RA who were SE + and ACPA + over six months in all four study cohorts. After adjusting for covariates that remained imbalanced between groups in the biologic-experienced PS-trimmed cohort, the improvement in CDAI score with abatacept versus TNFi was statistically significant. Given we found mostly numerical differences but not statistical significance for most of the comparisons, further studies are needed to explore these findings in a larger and more diverse patient population to fully explore the relationship between SE + status and differential response to medications.

## Electronic supplementary material

Below is the link to the electronic supplementary material.

Supplementary Material 1

Supplementary Material 2

## Data Availability

Data are available from CorEvitas, LLC through a commercial subscription agreement and are not publicly available. No additional data are available from the authors.

## Declarations

### Previous Presentation

A portion of this data was presented at ACR Convergence 2021, Virtual, November 5–9, 2021.
