# Supplementary material for: Comparative effectiveness of abatacept versus TNF inhibitors in rheumatoid arthritis patients who are ACPA and shared epitope positive
Source: Adv Rheumatol. 2024 Jan 19;64:10. doi: 10.1186/s42358-024-00352-4 (PMC13488744; doi:10.1186/s42358-024-00352-4)
Supplement: Supplementary file 1 [file 42358_2024_352_MOESM1_ESM.pdf]

Supplemental Figure 1. Study population flowchart

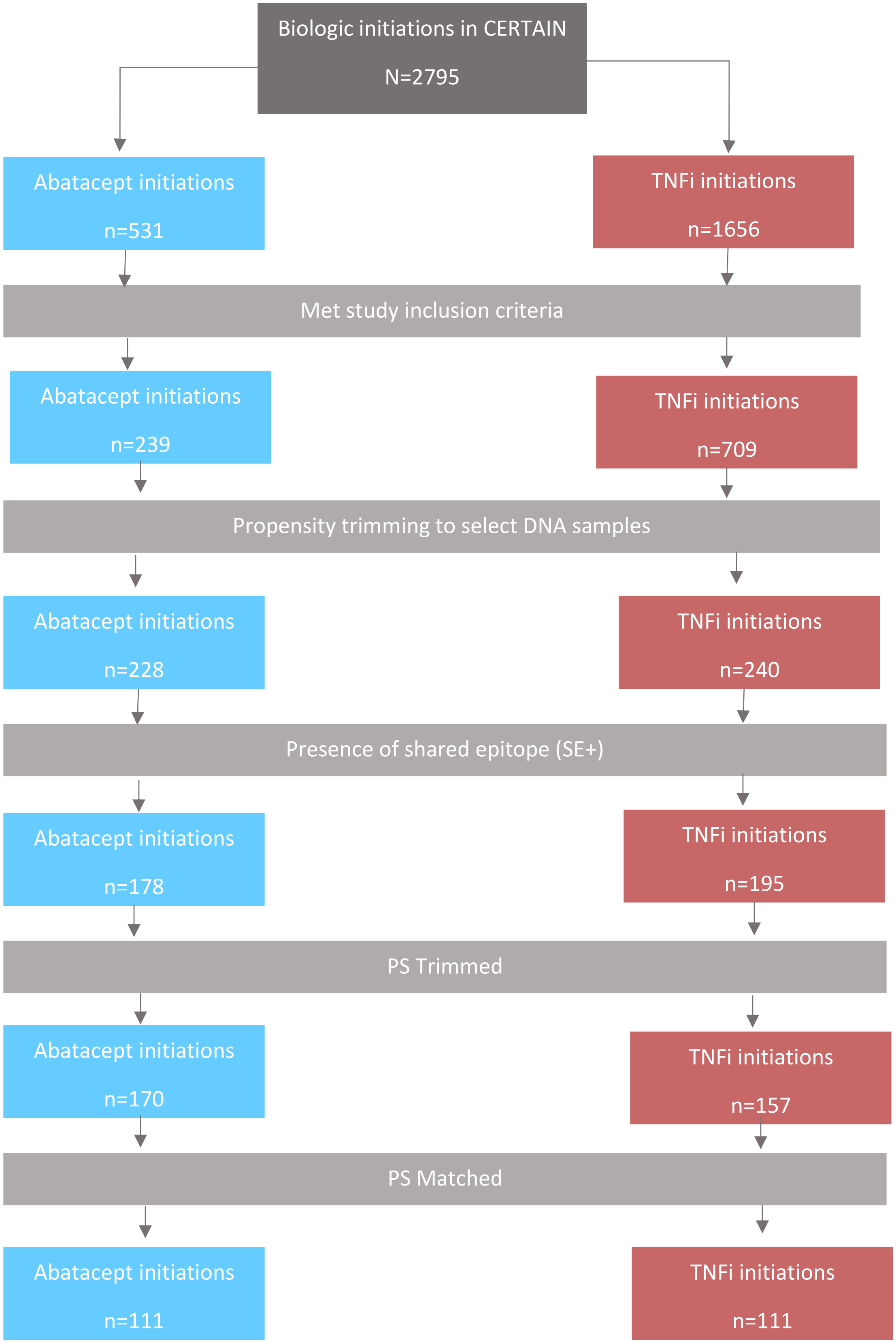

Supplemental Table 1. Patient demographic and clinical characteristics in SE+ abatacept and TNFi at baseline in total study cohort

At time of initiation	Abatacept<br>N=178	TNF<br>N=195	Standardized Difference[a]
Age (years), Mean (SD)	56.9 (12.9)	57.1 (13.1)	0.02
Female, n (%)	133 (74.7)	134 (68.7)	0.13
White, n (%)	172 (98.3)	179 (93.7)	0.23
Medicaid, n (%)	16 (9.0)	10 (5.1)	0.15
Full/Part time work, n (%)<br>Retired, n (%)<br>Disabled, n (%)	88 (49.4)<br>37 (20.8)<br>34 (19.1)	93 (47.7)<br>55 (28.2)<br>27 (13.9)	0.03<br>0.17<br>0.14
Never smoker, n (%)<br>Former smoker, n (%)<br>Current smoker, n (%)	72 (40.5)<br>59 (33.2)<br>47 (26.4)	79 (40.7)<br>72 (37.1)<br>43 (22.2)	0.01<br>0.08<br>0.10
BMI, Obese (≥30), n (%)	68 (38.2)	63 (32.3)	0.12
Hypertension, n (%)<br>Serious Infections, n (%)<br>Cardiovascular, n (%)	66 (37.1)<br>13 (7.3)<br>28 (15.7)	60 (30.8)<br>9 (4.6)<br>29 (14.9)	0.13<br>0.11<br>0.02
Duration of RA years, Mean (SD)	11.7 (10.5)	9.9 (10.5)	0.16
CDAI, Mean (SD)	28.3 (12.0)	28.6 (12.4)	0.03
TJC(28), Mean (SD)	10.2 (6.4)	10.1 (7.2)	0.01
SJC(28), Mean (SD)	7.7 (5.5)	8.2 (5.2)	0.09
CRP, Mean (SD)	10.3 (14.8)	11.6 (18.8)	0.08
DAS28-CRP, Mean (SD)	4.8 (1.1)	4.8 (1.0)	0.02
RF, Mean (SD)	193.6 (264.5)	233.3 (515.0)	0.10
RF+, n (%)	162 (91.0)	171 (87.7)	0.11
mHAQ, Mean (SD)	0.6 (0.5)	0.5 (0.5)	0.09
PGA (VAS 0-100),<br>Mean (SD)	49.0 (19.5)	52.0 (18.9)	0.16
PtGA (VAS 0-100),<br>PGA, Mean (SD)	54.8 (23.6)	50.5 (24.9)	0.18
Pain (VAS 0-100),<br>Mean (SD)	55.5 (24.4)	51.6 (28.1)	0.15
Fatigue (VAS 0-100),<br>Mean (SD)	56.4 (27.4)	50.9 (29.0)	0.19
Biologic-Naïve, n<br>Yes, n (%)	178<br>26 (14.6)	195<br>51 (26.2)	0.29
Prior csDMARD usage count,<br>Mean (SD)	178<br>1.3 (1.3)	195<br>1.0 (1.2)	0.19
Prior TNFi usage count, n<br>0, n (%)<br>1, n (%)<br>2+, n (%)	178<br>27 (15.2)<br>67 (37.6)<br>84 (47.2)	195<br>54 (27.7)<br>90 (46.2)<br>51 (26.2)	 0.31<br>0.17<br>0.45
Prior non-TNFi usage count, n<br>0, n (%)<br>1, n (%)<br>2+, n (%)	178<br>155 (87.1)<br>18 (10.1)<br>5 (2.8)	195<br>183 (93.9)<br>11 (5.6)<br>1 (0.5)	 0.23<br>0.17<br>0.18

At time of initiation	Abatacept N=178	TNF N=195	Standardized Difference[a]
Prior biologic usage count, n	178	195	
0, n (%)	26 (14.6)	51 (26.2)	0.29
1, n (%)	60 (33.7)	87 (44.6)	0.22
2, n (%)	57 (32.0)	46 (23.6)	0.19
3+, n (%)	35 (19.7)	11 (5.6)	0.43
Current MTX use, n	178	195	0.01
Yes, n (%)	90 (50.6)	98 (50.3)	
Current Prednisone use, n	178	195	0.05
Yes, n (%)	77 (43.3)	89 (45.6)	

[a]A standardized difference that is less than 0.10 indicates a negligible difference between treatment groups; therefore, variables with standardized differences greater than 0.10 between the groups were considered to have remained potentially unbalanced; BMI, body mass index; CDAI, Clinical Disease Activity Index; SD, standard deviation; TJC(28), Tender 28-Joint Count; SJC(28), Swollen 28-Joint Count; CRP, C-reactive protein; DAS28-CRP, Disease Activity Score-28 using CRP; RF, rheumatoid factor; mHAQ, modified Health Assessment Questionnaire; PGA, physician global assessment; PtGA, patient global assessment; VAS, visual analogue scale; csDMARD, conventional synthetic disease modifying anti-rheumatic drug; TNFi, tumor necrosis factor inhibitor; MTX, methotrexate
